# A case of anterior scleritis in association with posterior scleritis – a diagnostic riddle

**DOI:** 10.3205/oc000188

**Published:** 2022-02-08

**Authors:** Aditi Ghosh Dastidar, Sugandha Goel, Debi Kundu, Jyotirmoy Biswas, Eesh Nigam, Preeti Sharma

**Affiliations:** 1 Department of Cornea, Aditya Birla Sankara Nethralaya, Kolkata, West Bengal, India; 2 Department of Vitreo Retinal Services, Aditya Birla Sankara Nethralaya, Kolkata, West Bengal, India; 3 Department of Uvea and Ocular Pathology, Sankara Nethralaya, Chennai, Tamil Nadu, India; 4 Department of Uvea, Aditya Birla Sankara Nethralaya, Kolkata, West Bengal, India

**Keywords:** infectious scleritis, anterior scleritis, posterior scleritis, scleral abscess, exudative retinal detachment

## Abstract

We herein report a case of a young female presenting with multiple nodular scleral abscesses mimicking infective scleritis with exudative retinal detachment. Repeated diagnostic scraping for microbiological and histopathological analysis was inconclusive. The patient’s systemic and collagen disease work-up was non-contributory. She was treated with multiple surgical debridement and de-roofing of the abscesses along with antibiotic irrigation. Topical and oral steroid was stopped on worsening of the condition, and the patient was maintained on topical and oral antibiotics. Complete resolution of scleritis and exudative detachment was noted at 1 month follow-up and no recurrence was noted until 6 months.

## Introduction

Infectious scleritis is a rare but devastating condition [1], [2]. It can occur due to bacteria, fungus, protozoa, and virus [3]. It can be complicated by keratitis, cataract, glaucoma, endophthalmitis, exudative choroidal, and retinal detachment [1]. It is imperative to differentiate between immune and infectious to decide the line of treatment. Diagnosis and treatment of infectious scleritis becomes difficult in view of tunnel lesions in tightly bound collagen fibres of the sclera. We report a case of scleritis with exudative detachment and its successful management in spite of inconclusive microbiological and histopathological analysis.

## Case description

**Figure 1:**
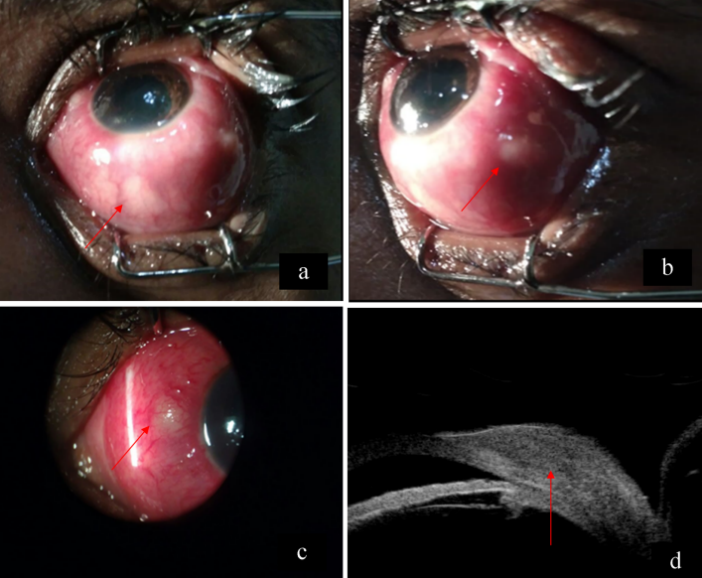
a–c) The left eye showing multiple scleral abscesses at presentation (arrows). d) Ultrasound biomicroscopy showing scleral thickening suggestive of scleritis with no underlying uveal tissue outpouching (arrow).

**Figure 2:**
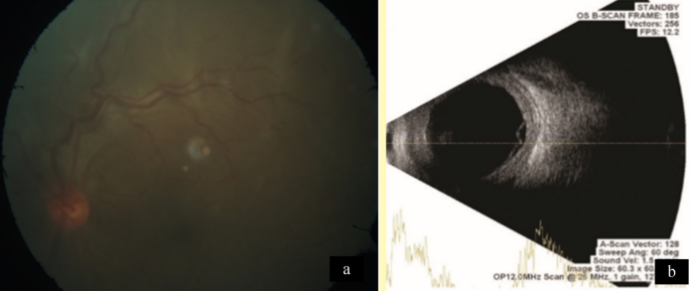
a) Fundus of the left eye showing exudative retinal detachment. b) B-scan ultrasonography showing exudative retinal detachment with thickened choroid and fluid in sub-Tenon’s space.

**Figure 3:**
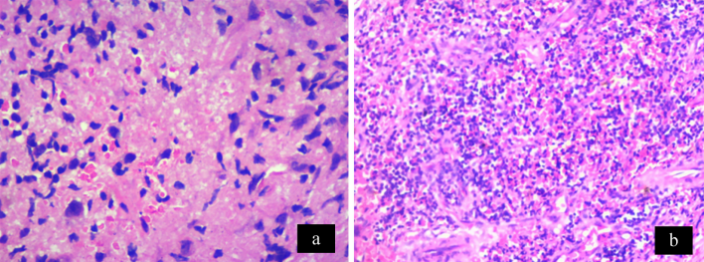
a) Histopathology of scleral scraping at presentation, and b) scleral biopsy at 1 week showing no organism with necrosis and focal collection of lymphocytes and plasma cells, suggestive of necrotizing granulomatous vasculitis.

**Figure 4:**
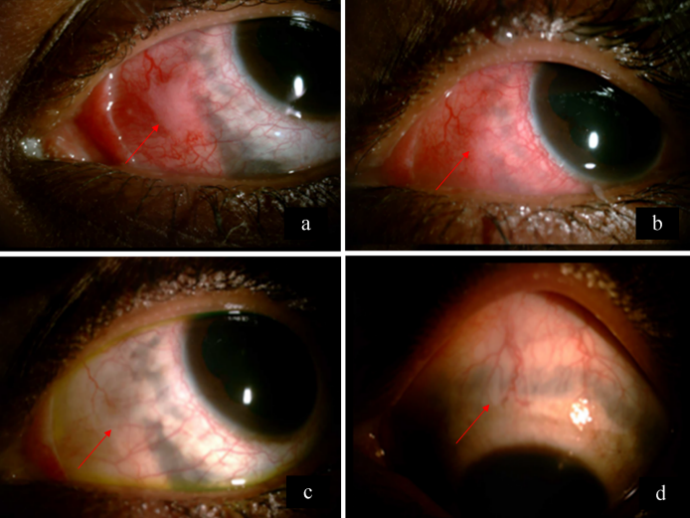
a) The left eye showing scleral abscess in the nasal quadrant with increased inflammation on 1 week follow-up after starting steroids (arrow). b) The scleral abscess is resolving on 2 weeks follow-up after stopping steroids (arrow). c) Complete resolution of the lesion (arrow) and d) scleral thinning is noted on 6 months follow-up (arrow).

A 28-year-old woman presented with redness, pain, and gradual diminution of vision in the left eye since 2 weeks. She was an agriculturist by occupation. She had no history of photophobia, ocular trauma, contact lens use, ocular surgery, fever, joint pain, rashes, Koch’s, contact, or any other systemic infection. There was no similar complaint in the past or in the other eye. On examination, the best corrected visual acuity (BCVA) in the right eye and the left eye was 6/6, N6, and 6/60, N12 respectively. Extraocular movements in the right eye were full. In the left eye, there was a mild limitation in abduction. The rest of the movements were full. Right-eye examination was unremarkable. Intraocular pressure was 12 mm Hg in both eyes. The left eye showed multiple scleral abscesses (Figures 1a–c (Fig. 1)) with anterior chamber cells 1+ and posterior synechiae. The cornea was not involved. Fundus of the left eye showed exudative retinal detachment (Figure 2a (Fig. 2)). B-scan ultrasonography showed exudative retinal detachment with thickened posterior ocular coat (choroid and sclera) and fluid in sub-Tenon’s space (Figure 2b (Fig. 2)). Ultrasound biomicroscopy prior to scraping in the left eye showed scleral thickening suggestive of scleritis (Figure 1d (Fig. 1)). A diagnosis of anterior and posterior scleritis with anterior uveitis was made. A diagnostic and therapeutic scleral scraping was done along with intraoperative antibiotic irrigation. Detailed microbiological testing was performed, including smear and culture. Smears underwent Gram staining, KOH mount, Giemsa staining and modified Ziehl-Neelsen acid fast staining to include all bacteria, fungus, and atypical micro-organism involvement. Culture for aerobic and anaerobic bacteria, Sabouraud dextrose agar for fungus, and Lowenstein- Jensen medium culture was done for acid fast organism. Microbiologic analysis showed no evidence of bacteria, fungus, or any atypical microorganism on smear and culture. The patient was started on topical (fortified tobramycin 1.4% and fortified cefazolin 5%) and systemic antibiotics (Ciprofloxacin); and topical steroids (Predacetate 1%) along with systemic non-steroidal anti-inflammatory drugs (NSAIDS) (Indomethacin). Histopathology (Figure 3a (Fig. 3)) showed the presence of necrotizing inflammation along with the presence of lymphocytes and plasma cells suggestive of necrotizing granulomatous vasculitis seen in polyangiitis or tuberculosis. In our case, we also noticed evidence of focal vasculitis surrounded by palisading histiocytes and lymphocytes. No microorganism was noted in the histopathological examination, thus suggesting a non-infectious etiology. The erythrocyte sedimentation rate (ESR) was raised (35 mm/hr). Mantoux test, anti-neutrophil cystoplasmic antibodies (P ANCA and C ANCA) and Quanti-FERON-TB gold test were negative. Polymerase chain reaction (PCR) from biopsied material for detection of eubacteria, panfungal and mycobacterial tuberculosis genome was negative. Chest X-ray was within normal limits. HLA B27 was negative. Hence the patient’s systemic and collagen disease work-up was also not supportive of histopathologic analysis. The patient was started on oral steroids (Prednisolone 1mg/kg/body weight weekly tapering) which led to worsening of the condition after 1 week (Figure 4a (Fig. 4)), suggesting infectious etiology, and thus both topical and oral steroids were stopped. Scleral biopsy was taken from the lesion in the nasal quadrant, which supported signs of inflammation and necrosis. No microorganisms were detected (Figure 3b (Fig. 3)). We continued weekly debridement with antibiotic irrigation and topical antibiotics, and the lesions started resolving (Figure 4b (Fig. 4)). Complete resolution of the abscesses was noted at 1 month (Figure 4c (Fig. 4)). BCVA in the left eye on 4 weeks follow-up improved to 6/9, N6, and after 6 months it was 6/6, N6. Scleral thinning was noted on 6 months follow-up with no evidence of recurrence (Figure 4d (Fig. 4)).

## Discussion

Scleritis is mostly idiopathic. Infectious scleritis accounts for only 5–10% of cases [4]. It presents as an ulcerated or non-ulcerated nodule. The clinical picture is similar to immune mediated scleritis. Around 30 to 50% of the non-infectious type of scleritis is associated with underlying autoimmune disease [5]. Autoimmune scleritis is characterized by varying mixtures of palisading granulomas, necrosis, and vasculitis. Infectious scleritis is characterized by acute inflammation and necrosis, and idiopathic scleritis is characterized by chronic non-specific inflammation with follicles and varying amounts of fibrosis [6]. A study by Riono WP et al. has documented nonzonal diffuse scleral inflammation similar to our case [7]. Pseudomonas aeruginosa is the most common causative agent in Western countries [8], [9], [10], [11]. However, fungus has been reported as the leading organism in developing countries like India [12]. Systemic steroids aid immune-mediated scleritis while they worsen infectious scleritis. Hence it is important to differentiate between these two conditions. In our case, the patient’s condition worsened on systemic steroids, suggesting infective etiology. Isolated infectious scleritis has a better prognosis than keratoscleritis. It is difficult to manage, as organisms remain in the tightly bound collagen fibres of the scleral layers which are poorly penetrated by the antimicrobials [13]. Surgical debridement aids as a diagnostic tool, therapeutically facilitates the penetration of antibiotics, and debulks the infected scleral tissue. Combined surgical debridement with intraoperative antibiotic irrigation and medical management shortens the course of treatment and produces a more favorable outcome. Ruling out infective etiology prior to administering steroids is a game changer. Material should be sent for both microbiological and histopathological analysis. PCR if available aids in ruling out mycobacterial etiology. Systemic and collagen disease work-up should be done to rule out immune etiology. Aggressive and prolonged topical and systemic antibiotics must be instituted in suspected cases of intrascleral dissemination [13]. In absence of perforation of the globe or severe scleral ectasia with uveal exposure during debridement, a scleral graft is not needed [1]. Probably exotoxin from the infectious organism is responsible for exudative retinal detachment. It may be a manifestation of severe infective scleritis, which can be well managed with surgical debridement of scleral abscess and aggressive antibiotic therapy.

## Conclusion

We conclude that infective etiology should be kept in mind in spite of non-contributory results of repeated scraping and surgical debridement. Scleral scraping should be sent for microbiological and histopathological analysis. Debridement is a diagnostic as well as a therapeutic aid. Scleral biopsy can be helpful in inconclusive cases to detect occult microorganisms in view of tunnel lesions seen in scleritis, and also to differentiate immune versus infective. Exudative retinal detachment in severe infective scleritis can be well managed with surgical debridement and antibiotic therapy.

## Notes

### Competing interests

The authors declare that they have no competing interests.
